# Mitophagy and Ferroptosis in Diabetic Kidney Disease: A Hypothesis of Their Synergistic Interplay in Renal Tubular Injury—A Narrative Review

**DOI:** 10.3390/ijms27198758

**Published:** 2026-09-30

**Authors:** Yanyu Gong, Qiuping Liu, Hanyuan Dai, Jialing Li, Xiulin Chang, Yudi Zhang

**Affiliations:** 1 School of Integrative Medicine, Chongqing College of Traditional Chinese Medicine, No. 61, Guoxue Road, Bicheng Street, Bishan District, Chongqing 402760, China; 2 Foundation Department, Chongqing Medical and Pharmaceutical College, No. 82, Daxuecheng Middle Road, Shapingba District, Chongqing 401331, China; 3 Chongqing Key Laboratory of Traditional Chinese Medicine for Prevention and Cure of Metabolic Diseases, Chongqing 402760, China

**Keywords:** diabetic kidney disease, renal tubular injury, mitophagy, ferroptosis

## Abstract

Tubular injury induced by mitophagy or ferroptosis serves as a critical pathological basis for the progression of diabetic kidney disease (DKD). Mitophagy constitutes a critical process of mitigating tubular injury by eliminating damaged organelles to maintain cellular equilibrium and homeostasis. Ferroptosis, a form of programmed cell death characterized by lipid peroxidation, serves as a pivotal factor in the progression of DKD. While numerous studies have elucidated the complex relationship between mitophagy and ferroptosis, there is a scarcity of literature addressing the relationship between these two processes in the context of tubular damage in DKD. We reviewed recent literatures exploring the associations among mitophagy, ferroptosis, and renal tubular injury in DKD, and tried to summarize the complex crosstalk between ferroptosis and autophagy in DKD. We hope to offer some new ideas for the treatment of DKD.

## 1. Introduction

Diabetic kidney disease (DKD), as one of the most common and serious microvascular complications in diabetes, is still a major problem in the global public health field, and its incidence in diabetic patients ranges from 25% to 40% [1,2]. Renal tubules, especially proximal tubules, play a central role in renal function, responsible for reabsorption of water, electrolytes and nutrients, and maintaining acid-base balance. Proximal tubule epithelial cells (PTECs) are one of the most energy demanding cells in the kidney, and highly rely on mitochondrial oxidative phosphorylation to meet their high Adenosine Triphosphate (ATP) requirements. Fatty acid β-oxidation (FAO) is the main energy source under physiological conditions [3,4]. In the context of DKD, however, this metabolic architecture renders PTECs uniquely vulnerable: chronic hyperglycemia drives the accumulation of Advanced Glycation End Products (AGEs) and lipotoxicity, while impairing FAO and mitochondrial function, leading to energy deficiency and cellular damage. This metabolic vulnerability renders tubular epithelial cells particularly susceptible to oxidative stress and cell death in the diabetic milieu [5,6]. In recent years, mitophagy and ferroptosis have each been independently implicated in the pathogenesis of renal tubular injury. Mitophagy, the selective autophagic clearance of damaged mitochondria, is essential for maintaining mitochondrial quality control in tubular epithelial cells, which are particularly susceptible to mitochondrial dysfunction due to their high energy demands [7]. In DKD, dysregulated mitophagy involving PTEN-induced kinase 1 (PINK1)/Parkin, Bcl-2-interacting protein 3 (BNIP3)/NIX (also known as Bcl2-interacting protein 3-like, BNIP3L), and FUN14 domain-containing protein 1 (FUNDC1) pathways is considered a critical driver of tubular injury, given that defective clearance of damaged mitochondria promotes oxidative stress and cell death [8]. Ferroptosis, an iron-dependent regulated cell death driven by lethal lipid peroxidation, has emerged as a key contributor to tubular epithelial cell loss in DKD. The susceptibility of renal tubular epithelial cells to ferroptotic damage is exacerbated by high glucose-induced oxidative stress and metabolic dysregulation, as reflected by iron overload, Glutathione Peroxidase 4 (GPX4) inactivation, and lipid peroxidation accumulation in diabetic kidneys and high glucose-treated tubular cells [9]. Of note, the interplay between mitophagy and ferroptosis has garnered considerable attention in other pathological settings, including hypoxic-ischemic brain injury, pancreatic cancer, and doxorubicin-induced cardiomyopathy [10,11,12].

However, their potential crosstalk specifically in the context of DKD remains largely unexplored. First, most existing reviews have discussed mitophagy and ferroptosis largely as separate pathways or in the context of non-renal diseases, with limited integration specifically within the DKD framework. Second, while individual studies have reported the involvement of each process in DKD-related tubular injury, a systematic synthesis that critically evaluates their convergence in renal tubular epithelial cells is lacking. Third, the temporal dynamics of their interaction across the course of DKD and the therapeutic implications of their dual targeting have not been comprehensively addressed. Based on the limitations and conflicting evidence in the current literatures, we propose the following core hypothesis: in DKD renal tubular epithelial cells, there may exist a bidirectional regulatory axis between mitophagy and ferroptosis—mitophagy may exert both “antagonistic” and “synergistic” effects on ferroptosis, with the balance between these two effects being dependent on disease stage, mitophagic intensity, and the cellular microenvironment. This review offers the following distinctive contributions: we systematically integrate the relationship between mitophagy and ferroptosis within the DKD framework, postulating the existence of a bidirectional regulatory axis—mitophagy may exert both antagonistic and synergistic effects on ferroptosis in DKD renal tubular injury, with the balance between the two effects potentially depending on disease stage, mitophagic intensity, and the cellular microenvironment. Based on this, we further propose a conceptual framework of “dynamic equilibrium-based intervention”: If the bidirectional regulatory hypothesis holds true, targeting the critical nodes governing the transition between antagonistic and synergistic effects may offer greater therapeutic potential than either promoting mitophagy or inhibiting ferroptosis alone. In contrast, their potential crosstalk specifically in the context of DKD remains largely unexplored.

## 2. Literature Search Strategy

Given that this article is a narrative review aimed at proposing the academic hypothesis of “synergistic crosstalk between mitophagy and ferroptosis in renal tubular injury in diabetic kidney disease,” rather than a systematic quantitative meta-analysis, the literature search strategy employed in this study balanced both systematicness and directedness. The databases searched included PubMed, Web of Science, EMBASE, Scopus, CNKI, Wanfang Data Knowledge Service Platform, and VIP Chinese Journal Service Platform. Full-text supplementation was additionally obtained through the official websites of SpringerLink, Elsevier ScienceDirect, and the Frontiers series of journals. The search timeframe was set from 2015 to 2026. For classic foundational literature in the fields of autophagy, ferroptosis, and mitochondrial dynamics, the timeframe was appropriately extended to incorporate key theoretical cornerstones.

The Chinese literature search employed a combination of Medical Subject Headings (MeSH) and free-text terms. The core MeSH terms included “diabetic nephropathy,” “diabetic kidney disease,” “mitophagy,” “ferroptosis,” “renal tubular injury,” “autophagy,” and “mitochondrial dysfunction.” Free-text keywords comprised “DKD,” “diabetic nephropathy (DN),” “selective autophagy,” “renal tubular epithelial cells,” “mitochondrial reactive oxygen species (mtROS),” “Nuclear factor erythroid 2-related factor 2 (Nrf2),” “GPX4,” “solute carrier family7 member11 (SLC7A11),” “PINK1/Parkin,” “BNIP3,” “FUNDC1,” “NLRP3 inflammasome,” “oxidative stress,” “renal fibrosis,” “iron metabolism,” “Adenosine Monophosphate-Activated Protein Kinase (AMPK),” and “Peroxisome proliferator-activated receptor gamma coactivator 1-alpha (PGC-1α).” English-language search terms included the MeSH headings “Diabetic Nephropathy,” “Diabetic Kidney Disease,” “Mitophagy,” “Ferroptosis,” “Renal Tubular Injury,” “Autophagy,” and “Mitochondrial Dysfunction,” supplemented with free-text keywords such as “DKD,” “DN,” “selective autophagy,” “renal tubular epithelial cell,” “mt ROS,” “Nrf2,” “GPX4,” “SLC7A11,” “PINK1/Parkin,” “BNIP3,” “FUNDC1,” “NLRP3 inflammasome,” “oxidative stress,” “renal fibrosis,” “iron metabolism,” “AMPK,” and “PGC-1α.” The core Boolean logic of the search strategy was constructed as (Diabetic Nephropathy OR Diabetic Kidney Disease) AND (Renal Tubular Injury OR Tubular Epithelial Cells) AND (Mitophagy OR Ferroptosis OR Mitochondrial Dysfunction), with appropriate modifications applied in accordance with the syntactic conventions of each individual database.

The inclusion criteria for the literature were as follows: (1) studies targeting diabetic kidney disease, renal tubular epithelial cells, or diabetic animal models; (2) studies addressing one or more aspects of mitophagy, ferroptosis, mitochondrial dysfunction, oxidative stress, and inflammatory mechanisms, with preferential inclusion given to studies that concurrently addressed the cross-regulatory interplay between mitophagy and ferroptosis; (3) studies encompassing cellular and animal-based fundamental experimental research, clinical observational studies, and mechanistic reviews; and (4) publications in either Chinese or English. The exclusion criteria comprised: (1) studies focusing exclusively on glomerular injury without involving tubular injury; (2) studies centered on neoplastic, neurological, or cardiovascular diseases without a direct relevance to renal tubular injury in diabetic kidney disease (a limited number of cross-disease mechanistic studies were retained to elucidate the core molecular pathways and interconnections between mitophagy and ferroptosis); (3) case reports lacking mechanistic exploration; (4) publications without available abstracts and duplicate publications.

## 3. Mechanism of Mitophagy in Renal Tubular Injury of DKD

Autophagy is a highly conserved self-degradation mechanism of cells under stress conditions. It selectively degrades damaged organelles (such as mitochondria) or abnormal proteins through the lysosomal pathway, thereby maintaining intracellular homeostasis [13]. Based on the mode of substrate transport, autophagy can be classified into three main types: macroautophagy, microautophagy, and chaperone-mediated autophagy [14]. Macroautophagy can be further classified into two distinct forms: non-selective and selective macroautophagy. Non-selective macroautophagy mediates bulk cytoplasmic degradation under nutrient deprivation conditions, whereas selective autophagy targets specific cargo (e.g., organelles or protein aggregates) via specialized cargo receptors; these receptors recognize their substrates and recruit them to the autophagosome membrane through the LC3-interacting regions (LIR) motif. The primary forms of selective autophagy include mitophagy (mitochondria), ferritinophagy (ferritin, mediated by Nuclear Receptor Coactivator 4,NCOA4), endoplasmic reticulum autophagy (endoplasmic reticulum, mediated by Family with sequence similarity 134, member B,FAM134B), and peroxisomal autophagy (peroxisomes) [15,16,17,18]. Among these, mitophagy has garnered particular attention in kidney diseases, owing to the high mitochondrial content and substantial metabolic demands of renal tubular epithelial cells. Unlike ferritinophagy, which targets ferritin via the cargo receptor NCOA4 to mobilize intracellular iron, mitophagy is uniquely defined by its cargo—the entire mitochondrion—and its dedicated recognition machinery [19,20]. Mitophagy is primarily achieved through ubiquitin-dependent and ubiquitin-independent pathways. In ubiquitin-dependent pathways, the PINK1/Parkin pathway is most extensively studied classical mechanism [21]. This pathway is initiated by the depolarization of mitochondrial membrane potential, which triggers PINK1 expression, subsequently recruits and activates the E3 ubiquitinligase Parkin, leading to ubiquitination of mitochondrial surface proteins. These ubiquitinated substrates are ultimately recognized and degraded by autophagosomes [22]. In contrast, the ubiquitin-independent pathway relies on LIR contained within mitochondrial outer membrane proteins (such as NIX/BNIP3L, BNIP3, and FUNDC1). These proteins can directly bind to the autophagy-related protein LC3, thereby bypassing the ubiquitination step and directly initiating the mitophagy process [23]. Although all three receptors (BNIP3, NIX/BNIP3L, and FUNDC1) engage the autophagy machinery via LIR-mediated LC3 binding, they are differentially regulated at the mechanistic level [24]. BNIP3 and NIX are primarily regulated at the transcriptional level: Hypoxia-inducible factor 1α (HIF-1α) upregulates their expression under hypoxic conditions [24,25]; under normoxic conditions, these proteins are restrained by SKP1-CUL1-F-box protein 4 (FBXL4) E3 ubiquitin ligase complex (SCF-FBXL4)-mediated ubiquitination and proteasomal degradation, which maintains their expression at low levels [26]. By contrast, FUNDC1 is predominantly regulated at the post-translational level: under normoxic conditions, Src kinase and Casein Kinase 2 (CK2) phosphorylate FUNDC1 at Tyr18 and Ser13, respectively, inhibiting its interaction with LC3 [27]. Moreover, Membrane-associated RING-CH-type finger protein 5 (MARCH5)-mediated ubiquitination of FUNDC1 serves as a negative feedback mechanism to prevent excessive mitophagy [25]. More critically, FUNDC1—unlike BNIP3 and NIX—can directly recruit the FAK family kinase-interacting protein of 200 kDa (FIP200)/UNC-51-like kinase 1 (ULK1) complex to initiate autophagosome formation, indicating a mechanistically distinct role in mitophagy initiation [24]. These mechanistic distinctions provide a conceptual framework for understanding how mitophagy is fine-tuned in response to diverse stress signals, and may have important implications for dissecting its context-dependent role in DKD renal tubular injury.

During the pathological progression of DKD, mitochondrial dysfunction-induced oxidative stress, inflammatory responses, and programmed cell death serve as critical factors for renal tubular injury [28]. Notably, among the DKD studies cited in this review, Han et al. employed chloroquine to verify autophagic flux, whereas other studies predominantly reported changes in mitophagy-related proteins (e.g., PINK1, Parkin, BNIP3, LC3-II, and sequestosome-1 (SQSTM1)), with limited direct evidence for autophagic flux [21]. This distinction is important because changes in protein levels may reflect transcriptional regulation or impaired degradation rather than genuine autophagic activity [21]. Restoring mitophagy-related protein expression through pharmacological or genetic interventions can significantly reduce production of Reactive oxygen species (ROS), inhibit cell apoptosis, and consequently alleviate renal tubular injury while improving renal function [29]. These findings provide a crucial theoretical foundation for targeted mitophagy-based therapeutic strategies for DKD. Nevertheless, the functional consequences of mitophagy in DKD tubular injury appear to be highly context-dependent, and its effects on oxidative stress, inflammation, and cell death are not always aligned.

### 3.1. Mitophagy Regulates Inflammatory Responses

Inflammatory responses act as exacerbating factors in the progression of DKD. Mitophagy plays a key role in modulating the onset and progression of inflammatory resoponses via multiple mechanisms. When mitochondria are damaged, they release oxidized and fragmented mitochondrial DNA (mtDNA) into the cytoplasm, which can activate the NLRP3 inflammasome, thereby triggering an inflammatory response [30]. In this case, effective mitophagy plays a protective role by clearing damaged mitochondria [31], thereby limiting the sources of mtDNA and ROS that promote inflammation. On the contrary, when the mitophagic flux in diabetic kidney is damaged or overwhelmed, the accumulation of damaged mitochondria will prolong the release of mtDNA and the production of ROS, thus creating a feed-forward loop, maintaining sterile inflammation and amplifying renal tubular damage [30,32]. Excessive ROS production further activates the inflammasome, and the enhanced inflammatory state promotes the formation of AGEs. When AGEs undergo impaired degradation or overproduction in renal tubules, they contribute to the progression of DKD [33]. These findings demonstrate that sustained inflammation and ROS production can lead to cellular apoptosis and necrosis, damaging renal tubular epithelial cells and further disrupting tubular structure and function. Conversely, the regulation of mitophagy can suppress excessive inflammatory responses, thereby exerting protective effects against renal inflammation under diabetic conditions [31]. Consistent with this notion, Xu et al. demonstrated that Prohibitin 2 (PHB2)-mediated mitophagy attenuates renal tubular epithelial cell injury by regulating mitochondrial dysfunction and NLRP3 inflammasome activation, further supporting the protective role of mitophagy in limiting NLRP3-driven inflammation [34]. Therefore, this seemingly paradoxical duality—whereby mitophagy can both suppress and fuel inflammation—likely reflects the differential engagement of distinct mitophagic pathways (e.g., PINK1/Parkin vs. receptor-mediated) and the context-dependent kinetics of mitochondrial clearance. In the early stages of DKD, efficient mitophagy may act as a rheostat to limit mtROS and mtDNA release; however, when mitophagic flux is overwhelmed or defective, the accumulation of damaged mitochondria can perpetuate a feed-forward loop of sterile inflammation and tubular injury [32]. Thus, rather than a simple on-off switch, mitophagy functions as a dynamic rheostat that fine-tunes the inflammatory milieu in DKD, and therapeutically restoring, rather than simply enhancing, its homeostatic capacity may be a more nuanced strategy.

### 3.2. Mitophagy as a Self-Amplifying Loop in Oxidative Stress-Mediated Renal Tubular Injury

In the process of DKD, the mitophagy defect of renal tubular epithelial cells and oxidative stress form a self amplifying pathological loop. Under physiological conditions, mitophagy mediated by PINK1/Parkin and BNIP3/NIX pathways maintains the dynamic balance between ROS generation and antioxidant defense by scavenging dysfunctional mitochondria [13,35]. As the main source of ROS in cells, mitochondria can produce excessive free radicals when their functions are damaged; While mitophagy can specifically clear dysfunctional mitochondria, reduce the generation of excessive ROS and inhibit the activation of mitochondria dependent apoptosis pathway [36,37].

In DKD, hyperglycemic conditions and metabolic disturbances can induce mitochondrial dysfunction, leading to excessive ROS production and release of pro-apoptotic factors (such as cytochrome c), and ultimately triggering apoptosis in renal tubular epithelial cells [34,38]. Meanwhile, excessive ROS accumulation and hyperglycemia further suppress key mitophagic pathways—including PINK1/Parkin, NIX/BNIP3, and FUNDC1—thereby impairing the clearance of damaged mitochondria. This establishes a self-amplifying “ROS–mitochondrial damage–mitophagy inhibition–more ROS” feed-forward loop: ROS suppress PINK1/Parkin- and BNIP3/NIX-mediated mitophagy, defective clearance of damaged mitochondria leads to further ROS release, which in turn exacerbates tubular injury and interstitial fibrosis [21,39]. Therefore, in-depth investigation of the context-dependent mechanisms of mitophagy in oxidative stress-mediated renal tubular injury, along with targeted regulation of key pathways such as PINK1/Parkin, NIX/BNIP3, or FUNDC1—particularly in a stage-specific manner—will not only help elucidate the molecular pathogenesis of DKD, but may also inform the development of more precise renal protective strategies. Importantly, targeting a single node within this loop may fail to achieve durable therapeutic efficacy; a combinatorial strategy that simultaneously enhances mitophagy, activates antioxidant responses, and stabilizes mitochondrial dynamics may offer more sustained protection against renal tubular injury in DKD. The molecular targets and their functions of the above-mentioned pathways are summarized in Table 1.

### 3.3. Mitophagy and Programmed Cell Death

Mitochondria, the core energy converters within cells, play a pivotal role in critical physiological processes, including metabolism, differentiation, growth, apoptosis, and death of cells [38]. As noted by Zhou et al. in their review, aberrant activation or dysfunction of mitophagy can lead to the release of large quantities of pro-apoptotic factors, thereby triggering cell death.

Apoptosis is the earliest recognized form of programmed cell death, and its intrinsic pathway is primarily regulated by mitochondria. B-cell lymphoma 2 (Bcl-2) family proteins participate in the initiation of apoptosis by regulating mitochondrial outer membrane permeabilization (MOMP), promoting cytochrome c release and caspase cascade activation [40]. Mitophagy can inhibit apoptosis by clearing damaged mitochondria and reducing the release of pro-apoptotic factors; conversely, mitophagic deficiency leads to the accumulation of damaged mitochondria, which amplifies apoptotic signals. In DKD renal tubular epithelial cells, high glucose-induced impairment of mitophagy has been confirmed to be closely associated with cellular apoptosis [41,42].

Necroptosis is a form of programmed necrosis mediated by the receptor-interacting protein kinase 1 (RIPK1)/receptor-interacting protein kinase 3 (RIPK3)/mixed lineage kinase domain-like protein (MLKL) pathway, and its activation is dependent on the phosphorylation of receptor-interacting protein kinases [43]. Mitophagy can inhibit necroptosis activation by clearing damaged mitochondria and reducing ROS production. When mitophagic function is impaired, excessive ROS production promotes the formation of the RIPK1/RIPK3 complex, thereby enhancing necroptotic signaling.

Pyroptosis is an inflammatory form of cell death mediated by inflammasome activation and caspase-1/11, characterized by gasdermin D (GSDMD) cleavage-induced plasma membrane pore formation and the release of pro-inflammatory cytokines, including interleukin-1β (IL-1β) and interleukin-18 (IL-18) [44]. Mitophagy can indirectly inhibit pyroptosis by clearing damaged mitochondria and reducing the release of mtROS and oxidized mtDNA, thereby suppressing excessive activation of the NOD-like receptor family pyrin domain-containing 3 (NLRP3) inflammasome [32].

Ferroptosis is an iron-dependent form of programmed cell death driven by dysregulated lipid peroxide metabolism, characterized fundamentally by disruption of intracellular redox homeostasis. As the primary site of cellular iron metabolism, mitochondria play a central role in the ferroptosis process [45,46]. Tatsuyori Morita et al. reported that dihydroartemisinin can induce ferroptosis by reducing mitochondrial transmembrane potential, and accelerating cellular iron autophagy and the generation of mtROS [47]. Moderate mitophagy can inhibit lipid peroxidation by clearing damaged mitochondria containing excessive free iron. However, excessive or insufficient mitophagy disrupts intracellular iron homeostasis, exacerbates lipid peroxide accumulation, and ultimately induces ferroptosis [28]. A series of studies based on animal models and cell experiments have shown that both upregulating the protein expression of the Sestrin2 (SESN2)/AMPK/PGC-1α axis and enhancing the activity of the Sestrin2/AMPK/PGC-1α axis, thereby delaying DKD progression and alleviating renal tubular injury [48,49]. On the other hand, dysregulated iron metabolism has been confirmed to exacerbate renal tubular injury in DKD. However, there is currently limited literature investigating the mechanistic pathways connecting mitophagy, ferroptosis, and renal tubular damage in DKD. Notably, studies have established interconnection between mitophagy and ferroptosis, specifically that aberrant mitophagy may lead to iron metabolism imbalance, thereby aggravating tubular injury. Therefore, targeting mitophagy to alleviate ferroptosis may imply a novel research direction and therapeutic strategy for DKD in the future.

In summary, mitophagy plays a critical regulatory role in apoptosis, necroptosis, pyroptosis, and ferroptosis by clearing damaged mitochondria and maintaining mitochondrial quality control. However, its functional outcomes are not fixed—under physiological conditions, moderate mitophagy protects cells from death, whereas in pathological microenvironments or when autophagic flux is impaired, mitophagy may shift from a “guardian” to an “executor”, promoting multiple forms of programmed cell death [38].

Among the four death pathways discussed above (Table 2), ferroptosis is of particular interest due to its unique association with mitochondrial iron metabolism and lipid peroxidation. Accumulating evidence indicates that ferroptosis in DKD renal tubular injury is driven by multiple mechanisms: inflammatory responses promote ferroptosis through pro-inflammatory cytokine release and lipid peroxidation [50]; oxidative stress directly disrupts the GPX4/SLC7A11 antioxidant system, compromising the lipid peroxidation defense barrier [51]; and mitochondrial dysfunction directly provides both iron sources and ROS for ferroptosis [28]. These three factors do not operate in isolation but rather interact and synergistically amplify each other, collectively driving ferroptosis in DKD tubular epithelial cells [52]. In the following sections, we will systematically discuss the molecular mechanisms and regulatory strategies of ferroptosis in DKD renal tubular injury from the perspectives of inflammatory responses, oxidative stress, and mitochondrial dysfunction.

## 4. Mechanism of Ferroptosis in Renal Tubular Injury of DKD

As an emerging form of regulated cell death, the mechanisms of ferroptosis in various diseases are being progressively elucidated. In experimental studies, this is typically validated through the following approaches: cell death can be effectively reversed by ferroptosis-specific inhibitors (such as Ferrostatin-1, Liproxstatin-1) or iron chelators (such as Deferoxamine) [53]. Ferroptosis is also accompanied by downregulation of GPX4 and SLC7A11, upregulation of acyl-CoA synthetase long-chain family member 4 (ACSL4), GSH depletion, and accumulation of lipid peroxidation end products (such as malondialdehyde, MDA, and 4-hydroxynonenal, 4-HNE) [54]. lipid peroxidation levels can be detected using the c and typical morphological changes—including mitochondrial shrinkage and cristae reduction—can be observed under electron microscopy [53,55]. Of note, in addition to being modulated by mitophagy, ferroptosis can also be triggered through multiple autophagy-independent mechanisms—either by direct inhibition of the SLC7A11/glutathione(GSH)/GPX4 axis or by non-autophagic iron overload [48,49,56]. However, the ferroptosis discussed in the following sections is largely related to mitophagy. In the context of kidney injury, the role of ferroptosis has been extensively investigated, and its close association with renal tubular injury in DKD involves mechanisms such as inflammatory responses, oxidative stress, and mitochondrial dysfunction [49,50,51].

### 4.1. Inflammatory Response-Induced Ferroptosis

Inflammation is a defensive response of the organism to injury or infection, which helps maintain tissue homeostasis. However, excessive inflammatory responses can cause harm to the body. Studies on the machanisms of inflammation have shown that activated immune cells can release large amounts of ROS and pro-inflammatory cytokines, thereby inducing oxidative stress and cytotoxicity, ultimately leading to tissue injury [57]. Furthermore, aberrant inflammatory responses are closely associated with dysregulated iron metabolism and imbalanced redox system. Pro-inflammatory cytokines such as IL-1β, interleukin-6 (IL-6), Tumor Necrosis Factor-α (TNF-α), and Interferon-gamma (IFN-γ) can modulate ferritin synthesis, thereby influencing intracellular iron storage and metabolism [58]. Clinical studies have shown that serum levels of IL-1β, IL-6 and TNF-α are significantly positively correlated with the ferroptosis driver proteins Arachidonate 15-Lipoxygenase (ALOX15) and Heme Oxygenase 1 (HO-1) in patients with DKD (all *p* < 0.001), suggesting a synchronization between the degree of inflammation and ferroptosis activation in DKD [59]. Mechanistically, IL-6 can upregulate hepcidin expression through activation of the Janus kinase 2/signal transducer and activator of transcription 3 (JAK2/STAT3) signaling pathway, promoting the internalization and degradation of ferroportin, thereby limiting the iron efflux capacity of renal tubular cells and leading to intracellular free iron accumulation [60]. Meanwhile, upregulation of iron import proteins such as divalent metal transporter 1 (DMT1) has also been observed in diabetic renal tubular epithelial cells, which may be associated with the action of inflammatory factors such as TNF-α, further exacerbating intracellular iron overload [61]. The iron metabolic reprogramming induced by the aforementioned pro-inflammatory cytokines—increased iron uptake coupled with blocked iron efflux—may constitute a metabolic prerequisite for ferroptosis, providing a molecular explanation for the functional coupling between inflammation and ferroptosis. Given that ROS accumulation and dysregulated iron metabolism are key drivers of ferroptosis, it is plausible that a close interconnection exists between inflammatory responses and ferroptosis.

Recent studies have further elucidated the mechanisms by which inflammatory responses induce ferroptosis. IFN-γ suppresses the Janus kinase 1/signal transducer and activator of transcription 1/solute carrier family 7 member 11 (JAK1–STAT1–SLC7A11) signaling axis, thereby reducing the synthesis of GSH—a key antioxidant that inhibits ferroptosis [62], excessive accumulation of intracellular ferrous ions (Fe^2+^) activates the NLRP3 inflammasome via the Cyclic GMP-AMP Synthase–Stimulator of Interferon Genes 1 (cGAS–STING1) pathway, which exacerbates oxidative stress and promotes lipid peroxidation, ultimately culminating in ferroptosis is noteworthy that the NLRP3 inflammasome plays a critical role in renal pathologies characterized by proteinuria [63], such as in DKD, contributing to glomerular and tubular injury, as well as fibrosis [64]. Therefore, in-depth investigation into the interplay between inflammatory responses and ferroptosis in the progression of DKD may reveal novel therapeutic targets for renal tubular damage.

### 4.2. ROS-Induced Ferroptosis

Under physiological conditions, ROS are generated through enzymatic and non-enzymatic pathways and exert limited effects on cellular functions. However, when ROS levels exceed the physiological threshold, they can react with cellular components such as DNA, lipids, and proteins, leading to oxidative damage and cellular dysfunction [65]. In recent years, the role of ROS in renal tubular injury has been gradually elucidated. Excessive ROS production can activate uncoupling protein 2 (UCP2) in the kidneys, leading to dissipation of the proton driving force in the inner mitochondrial membrane in the form of heat energy, reduced ATP synthesis, and impaired energy supply in renal tubular cells, hence exacerbating tubular injury [66]. Moreover, UCP2 has been shown to mitigate apoptosis of renal tubular epithelial cells in acute kidney injury by reducing ROS generation supporting the critical role of ROS in tubular damage [67]. Additionally, ROS can attack polyunsaturated fatty acids, triggering lipid peroxidation, generating products such as malondialdehyde (MDA), disrupting cell membrane integrity and ultimately inducing ferroptosis [68,69].

Studies based on cell experiments have demonstrated that high glucose-cultured human renal proximal tubular epithelial cells (HK-2) exhibit significantly elevated levels of ROS and MDA, along with characteristic morphology of ferroptosis, such as mitochondrial shrinkage, increase in double-membrane density, and reduction of mitochondrial cristae [70]; furthermore, ferroptosis inhibitors have been shown to markedly ameliorate high glucose-induced renal tubular injury [48]. As a critical antioxidant, GSH suppresses ferroptosis by reducing ROS generation, further underscoring the central role of ROS in the regulation of ferroptosis [71]. Therefore, reducing ROS accumulation to attenuate ferroptosis may suggest a promising therapeutic strategy for mitigating renal tubular injury in DKD.

### 4.3. Mitochondrial Dysfunction-Induced Ferroptosis

The kidney, an organ with high mitochondrial abundance, relies heavily on the maintenance of mitochondrial homeostasis for its proper function. Disruption of mitochondrial dynamics—such as imbalanced fusion and fission, impaired autophagy, and oxidative stress—can lead to mitochondrial dysfunction. Such dysfunction is implicated in the pathogenesis of various renal diseases, including DKD, and has recently been demonstrated to play a critical role in renal tubular injury in DKD [41,72].

Furthermore, recent studies based on the relationship between mitochondria and ferroptosis have revealed a significant overlap in the mechanisms underlying mitochondrial dysfunction and ferroptosis, particularly in terms of ROS generation and lipid peroxidation. Notably, alteration in mitochondrial ultrastructure characterizes ferroptotic cells [39]. In addition, certain pharmacological agents, such as polyphyllin I and lapatinib, have been shown to induce mitochondrial dysfunction through distinct pathways and to promote ferroptosis in experimental studies [73]. In diseases including cancer, the regulation of mitophagy has also been demonstrated to be associated with the occurrence of ferroptosis [11,74]. In DKD, overexpression of canopy FGF signaling regulator 2 (CNPY2) has been found to accelerate ferroptosis and disrupt mitochondrial-associated endoplasmic reticulum membrane (MAM) integrity, leading to mitochondrial dysfunction and endoplasmic reticulum stress, thereby aggravating renal tubular injury in DKD [72]. Conversely, preserving mitochondrial function and inhibiting ferroptosis have been shown to provide renal protection and attenuate injury [75]. These findings suggest that mitochondrial dysfunction may influence ferroptosis and participate in the mechanisms of renal tubular injury in DKD.

Both mitophagy and ferroptosis have been demonstrated to play critical roles in the progression of DKD, and their close interrelationship offers a novel perspective for investigating the pathogenesis of DKD. Impaired mitophagy may contribute to renal tubular injury, with ferroptosis potentially acting as one of its downstream effector mechanisms. Therefore, in-depth research into the interplay between mitochondrial dysfunction and ferroptosis in DKD will not only enhance the understanding of its pathological mechanisms but may also provide a theoretical foundation for developing new therapeutic strategies. The molecular targets and their functions of the above-mentioned pathways are summarized in Table 3.

## 5. Interplay Between Mitophagy and Ferroptosis in Renal Tubular Injury

**Figure 1:**
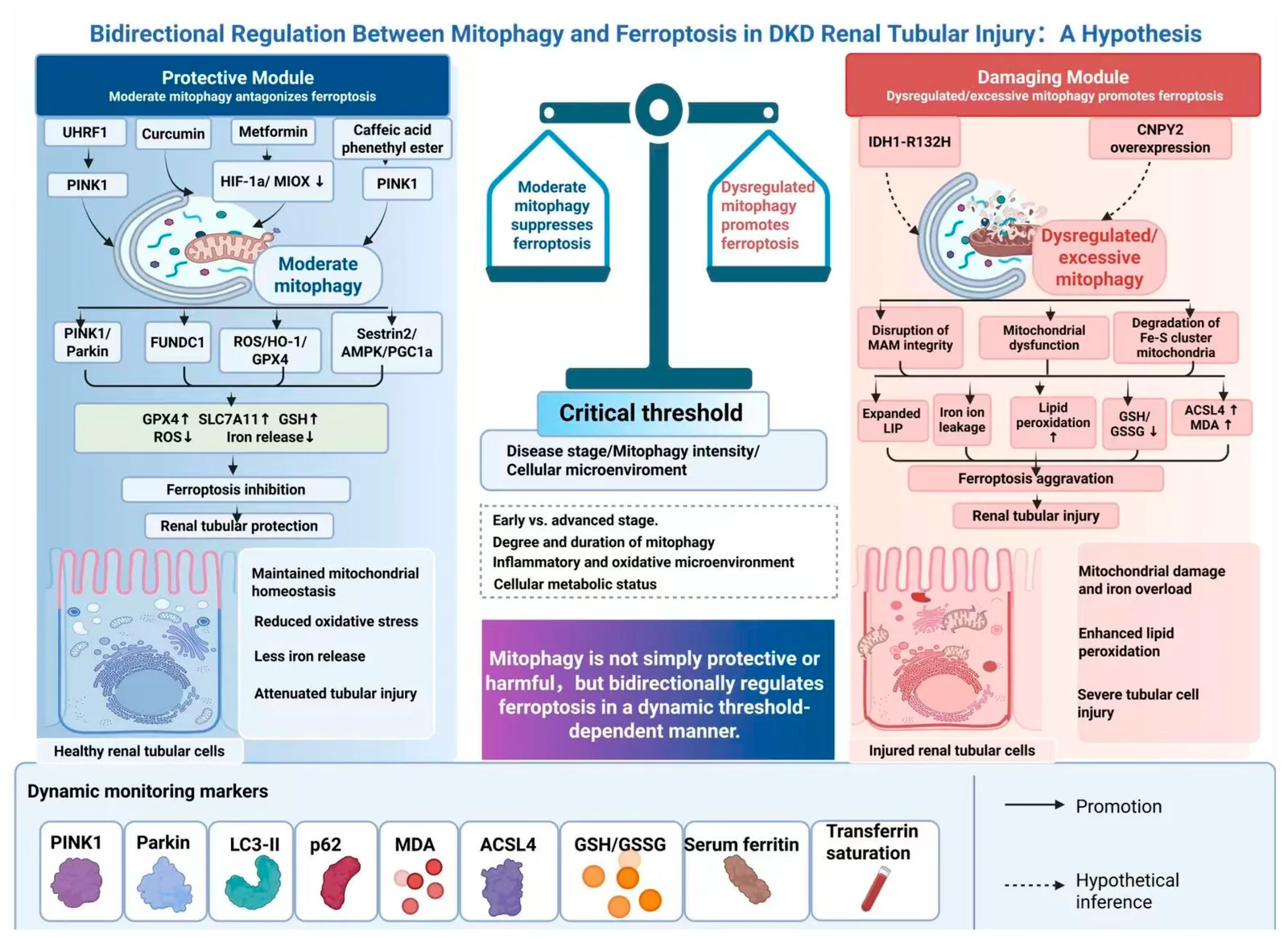
Bidirectional regulation between mitophagy and ferroptosis in renal tubular injury of diabetic kidney disease (DKD): a hypothesis. The protective module indicates that moderate mitophagy antagonizes ferroptosis and exerts renoprotective effects on renal tubules [77,78,79,80]; the injury module indicates that dysregulated mitophagy may promote ferroptosis and aggravate renal tubular injury [72,81]. A critical threshold may exist between these bidirectional effects, and this threshold is influenced by factors such as disease stage, mitophagy intensity, and the cellular microenvironment. Meanwhile, the figure summarizes some key dynamic monitoring biomarkers of this bidirectional regulatory process. Solid arrows represent promoting effects; dashed arrows represent hypothetical inferences.

Mitophagy exerts dual roles in the modulation of ferroptosis (Figure 1). Under mild stress or in the initial phases of iron overload, mitophagy can potentially inhibit ferroptosis through the elimination of dysfunctional mitochondria and the sequestration of iron within these organelles. This process consequently reduces the levels of ROS production and mitigates oxidative stress. Nevertheless, excessive autophagic activity due to dysfunctioning mitophagy may significantly increase iron release and facilitate lipid peroxidation, ultimately aggravating ferroptosis [76]. At present, this dynamic equilibrium mechanism has been predominantly substantiated in isolation within other disease models, where it has undergone relatively systematic validation. However, it is noteworthy that, within the context of metabolic dysregulation characteristic of DKD, renal tubular epithelial cells—being parenchymal cells endowed with high mitochondrial density and active iron metabolism—remain uncertain as to whether they conform to analogous regulatory principles. To date, no direct evidence has conclusively established such a relationship in this specific setting, with only a few studies hinting at potential cross-talk between these processes in renal injury models. On the basis of available experimental data, we therefore propose an integrated hypothesis positing a latent interplay during the pathogenesis of tubular injury in DKD (Figure 1). This conceptual framework is intended to open novel avenues for future interventional strategies targeting DKD-associated tubular damage and to address the existing void in knowledge within this field.

### 5.1. Antagonistic Effects of Mitophagy and Ferroptosis in Renal Tubular Injury

Both mitophagy and ferroptosis play significant roles in renal tubular injury, and their interplay has been extensively discussed in recent years. In chemically induced models of renal injury, mitophagy has been shown to attenuate tubular cell ferroptosis and renal damage through the ROS/HO-1/GPX4 axis [82]. Additionally, mitophagy can ameliorate tubular injury via the Sestrin2/AMPK/PGC-1α pathway, thereby restoring mitochondrial function and suppressing ferroptosis [49]. In the aforementioned disease models, the antagonistic relationship between mitophagy and ferroptosis within the mechanisms of renal tubular injury is vividly illustrated, wherein mitophagy confers renoprotective effects by inhibiting ferroptosis. In the DKD model of renal injury, which constitutes the primary focus of this review, such mechanisms have likewise been substantiated. Ubiquitin-like with PHD and ring finger domains 1 (UHRF1), a critical epigenetic regulator functioning as a molecular bridge connecting DNA methylation and histone modification, confers protection against high glucose-induced ferroptosis in HK-2 cells by promoting PINK1-mediated mitophagy, thereby alleviating renal injury [77]. Furthermore, curcumin has been demonstrated to re-activate mitophagy—which is suppressed under high-glucose conditions—thereby protecting renal tubular cells from ferroptotic damage [78]. Metformin has been shown to downregulate the HIF-1α/myo-inositol oxygenase (MIOX) signaling axis in renal tubular epithelial cells, consequently restoring mitophagy and inhibiting ferroptosis [79]. In addition, mechanistic investigations into the protective effects of caffeic acid phenethyl ester against tubular injury in DKD have revealed that this compound upregulates the expression of GPX4 and SLC7A11 in high glucose-stimulated cells, elevates GSH levels, and suppresses excessive generation of lipid hydroperoxides induced by high glucose, thereby mitigating ferroptosis. This effect was demonstrated to be dependent on PINK1-mediated mitophagy through knockdown of PINK-1 using small interfering RNA [80]. Collectively, these findings indicate that certain pharmacological agents or regulatory factors may act upon the crosstalk mechanisms between mitophagy and ferroptosis, exerting renoprotective effects of considerable significance in DKD by restoring or moderately enhancing mitophagy, eliminating dysfunctional mitochondria, reducing ROS release, and inhibiting ferroptosis [83]. The detailed mechanism is shown in the protective module (Figure 1). In conclusion, an antagonistic interaction between mitophagy and ferroptosis has been corroborated within the pathogenic mechanisms of tubular injury in DKD; nevertheless, the specific molecular underpinnings warrant further elucidation. Future investigations delving deeper into this domain are merited, with the aim of providing more diversified therapeutic avenues for DKD-associated tubular injury. An overview of the above-mentioned frontier studies is presented in Table 4.

### 5.2. Synergistic Effects of Mitophagy and Ferroptosis in Renal Tubular Injury

NRF2, as an antioxidant transcription factor, participates in the regulation of mitochondrial function and influences mitochondrial homeostasis. Knockdown of NRF2 alters mitochondrial function and further modulates ferroptosis, suggesting a potential mechanistic crosstalk between mitochondrial function and ferroptosis that synergistically contributes to disease pathogenesis [52]. This association, however, is not unidirectional. Investigations in the oncology field have demonstrated that mitophagy promotes the degradation of mitochondria rich in iron-sulfur clusters, resulting in iron ion release and expansion of the labile iron pool (LIP). When mitophagy is excessively activated, increased lysosomal iron content may lead to iron leakage, subsequently triggering lipid peroxidation and ferroptosis [11]. Beyond its extensive discussion in cancer models, this potential mechanism also holds relevance in models of drug-induced renal tubular injury. For instance, in a patulin-induced acute kidney injury model based on animal experiments and human renal tubular epithelial cell assays, pharmacological blockade of autophagy with 3-methyladenine (3-MA) significantly attenuated ferroptosis, implying a possible causal relationship between autophagy and ferroptosis [84], wherein autophagy may, under certain contexts, act as an upstream driver of ferroptosis. Concurrently, the Isocitrate Dehydrogenase 1—Arginine to Histidine mutation at position 132 (IDH1-R132H) mutation has been shown to exacerbate mitochondrial lipid peroxidation and dysfunction in renal tubules, augmenting cisplatin-induced oxidative stress and ferroptosis, thereby promoting kidney injury [81]. Collectively, these findings indicate that aberrant mitophagy may alter systemic oxidative stress and iron release profiles, thereby influencing ferroptosis onset, with both processes synergistically exacerbating renal damage (Figure 1). Although such synergistic relationships have been corroborated in other pathological models (Table 5), whether a similar paradigm exists in DKD—the specific focus of our inquiry—warrants investigation. In the unique metabolic milieu of DKD, preliminary evidence already supports this hypothesis. Overexpression of CNPY2 disrupts MAM integrity, induces mitochondrial dysfunction, and is accompanied by heightened ferroptosis, with both processes synergistically aggravating tubular injury in DKD [72].

Mitophagic dysfunction occupies a critical position in the progression of tubular injury in DKD. Ferroptosis, in turn, can be regulated via the PINK1/Parkin-mediated ubiquitination pathway or through receptor-mediated pathways involving FUNDC1 [22,85]. Existing evidence indicates that, in certain specific renal injury models, mitophagy may synergize with ferroptosis to promote tubular damage [42,86]; conversely, under certain circumstances in DKD, restoration or enhancement of mitophagy may protect renal tubules by suppressing ferroptosis. Nevertheless, direct evidence elucidating the mechanisms underlying the progression of tubular injury in DKD remains lacking—both antagonistic and synergistic effects of mitophagy and ferroptosis coexist in DKD, yet the critical conditions governing the transition between these two modalities cannot be delineated at present. On this basis, we propose the following hypothesis (Figure 1): during the progression of tubular injury in DKD, a complex interplay may exist between mitophagy and ferroptosis. On one hand, extensive mitophagy resulting from mitochondrial dysfunction may synergize with ferroptosis to promote tubular damage [42,72,81]. On the other hand, moderate mitophagy may alleviate oxidative stress, reduce ROS levels, and inhibit ferroptosis, thereby counteracting its deleterious effects on renal tubules [80,87,88]; a critical threshold conditions the transition between these two outcomes. Should this hypothesis be validated, it would confer greater therapeutic precision than merely inhibiting ferroptosis or promoting mitophagy in isolation.

In summary, within the realm of investigations into the interplay between mitophagy and ferroptosis, these seemingly contradictory observations ultimately converge toward a unified conclusion: the regulatory effect of mitophagy on ferroptosis is not intrinsically pro- or anti-ferroptotic, but rather is highly contingent upon disease type, autophagic intensity, and cellular context (Figure 1). Further exploration of the bidirectional relationship between these processes, alongside identification of the dynamic equilibrium point bridging autophagic intensity and the ferroptotic threshold, will be of substantial significance for future strategies aimed at mitigating tubular injury in DKD.

## 6. Conclusions and Perspectives

This review systematically elucidates the roles of mitophagy and ferroptosis in DKD-associated tubular injury from multiple perspectives, and explores their potential interrelationship. Synthesizing the available evidence, we propose the following core hypothesis (Figure 1): during the progression of tubular injury in DKD, a potential bidirectional regulatory axis exists between mitophagy and ferroptosis. In the early stages of the disease or under conditions of mild cellular stress, mitophagy exerts a protective role by clearing damaged mitochondria, alleviating oxidative stress, reducing ROS levels, and limiting iron release, thereby antagonizing ferroptosis and preserving tubular integrity [4,5]. Under certain pathological circumstances, however, dysregulated mitophagy may lead to augmented iron release and disruption of the lipid peroxidation barrier, thereby converting into a synergistic factor that exacerbates tubular damage in concert with ferroptosis [72]—at which point mitophagy paradoxically becomes a facilitator of ferroptosis, establishing a vicious cycle. Thus, mitophagy may exert dual—both antagonistic and synergistic—effects on ferroptosis in DKD-associated tubular injury. We postulate that the critical juncture governing this switch may be associated with modulatory factors of mitophagy, potentially including the activation status of PINK1/Parkin and FUNDC1 pathways, the extent of MAM integrity disruption, or cellular environmental conditions. At present, although indirect evidence from other disease models and selected DKD models lends support to this hypothesis, direct proof demonstrating the coexistence of both effects remains insufficient, and the conditions governing the transition between these two modalities remain obscure—constituting a significant knowledge gap in current research.

Given that the majority of existing studies have been conducted in non-renal models or in renal injury models induced by other etiologies, with only limited investigations supporting their applicability to DKD, further validation is urgently required. We therefore recommend that future research focus on the following aspects. First, dynamic monitoring of mitophagy and ferroptosis markers across different stages of DKD (e.g., microalbuminuria, macroalbuminuria, and renal failure) should be undertaken. Based on the mechanisms discussed in this review, we propose prioritizing the following categories of candidate biomarkers. The levels of PINK1, Parkin, LC3-II, and p62 can reflect mitophagic activity in diabetic kidney disease (DKD) [89,90]. Indicators such as MDA, ACSL4, the GSH/GSSG ratio, and parameters related to the labile iron pool—including serum ferritin and transferrin saturation—may serve as indirect estimators of ferroptotic burden [91,92]. A combined assessment of these two biomarker panels, formulated as a “mitophagy–ferroptosis imbalance index,” could help validate the presence of a dual effect and enable dynamic monitoring of the critical transition point from antagonism to synergy between these two processes. Notably, renal tubular injury in early-stage DKD often progresses insidiously. The development of highly sensitive urinary or blood-based biomarkers is therefore of considerable importance for identifying high-risk patients or tracking disease evolution before irreversible tubular damage ensues. In this context, particular attention should be directed toward serum ACSL4, serum ferritin, and the mRNA expression levels of PINK1 and Parkin [89,91]. Furthermore, Regucalcin (RGN), also known as senescence marker protein-30 (SMP30), is a multifunctional calcium-binding protein; studies have shown that the expression levels of regucalcin and cluster of differentiation 63 (CD63), among other molecules in urinary exosomes, may serve as potential biomarkers for DKD [93]. Such findings may herald a novel and promising avenue for future biomarker exploration; and combined detection of these two categories of biomarkers to construct a “mitophagy-ferroptosis imbalance index,” which may facilitate validation of the dual effects and enable dynamic monitoring of the critical transition point from antagonism to synergy. Second, in-depth investigation into the key determinants governing the transition between these two effects is warranted. Should the critical node be identified, further exploration of the therapeutic potential of interventions targeting this transition point—through appropriately designed experiments in animal models or cellular systems—would be essential to evaluate both efficacy and safety of such strategies.

In recent years, therapeutic strategies targeting the mechanistic pathways of mitophagy and ferroptosis have demonstrated preliminary potential in protecting against tubular injury in DKD. Classical ferroptosis inhibitors, including Ferrostatin-1, Liproxstatin-1, and GPX4 agonists, have exhibited protective effects across various kidney disease models, and in vitro experiments have confirmed their capacity to significantly ameliorate the characteristic ferroptotic alterations in high glucose-induced renal tubular epithelial cells [69,70]. Concurrently, the renoprotective actions of certain pharmaceutical agents and natural bioactive compounds, functioning as mitophagy agonists in the context of tubular injury, have been elucidated. Metformin restores mitophagy via the HIF-1α/MIOX signaling axis, thereby inhibiting ferroptosis in renal tubular epithelial cells [4]. Caffeic acid phenethyl ester, dependent upon PINK1-mediated mitophagy, upregulates the expression of GPX4 and SLC7A11, consequently attenuating ferroptosis in these cells [5]. Curcumin reverses the suppression of mitophagy, protecting renal tubular epithelial cells from ferroptotic damage [78]. Nevertheless, in light of our proposed hypothesis—that a potential bidirectional dynamic regulatory axis exists between mitophagy and ferroptosis, with both antagonistic and synergistic effects coexisting during the progression of tubular injury in DKD, and that the equilibrium between these processes may be contingent upon disease stage, autophagic intensity, and cellular microenvironment—mere promotion of mitophagy or inhibition of ferroptosis in isolation may not represent the optimal strategy. Rather, an ideal intervention should target the critical factors governing the transition between the two effects, aiming to modulate mitophagy to a “functionally appropriate” dynamic equilibrium range, thereby achieving more stable therapeutic outcomes.

In summary, in contrast to previous reviews, we have systematically integrated the relationship between mitophagy and ferroptosis in DKD-associated tubular injury and proposed a novel hypothesis of “bidirectional dynamic regulation” between these two processes. This conceptual framework not only deepens our multidimensional understanding of the complex pathological mechanisms underlying DKD, but also provides a theoretical foundation for the development of dynamic equilibrium-based intervention strategies targeting this bidirectional regulatory axis. Nevertheless, several limitations of this review should be acknowledged. First, research on the interplay between mitophagy and ferroptosis in DKD remains in its nascent stages, with direct evidence being relatively limited; certain conclusions are primarily extrapolated from studies in other disease models or acute kidney injury, and their applicability to the chronic metabolic milieu of DKD awaits further confirmation. Second, systematic experimental data supporting the critical conditions and regulatory mechanisms governing the transition between the two effects are currently lacking, and the definitive validation of the aforementioned hypothesis will require higher-level evidence in future investigations.

## Figures and Tables

**Table 1:** Major roles of Mitophagy in diabetic nephropathy.

Key Molecules/Targets	Roles	Mechanisms
PHB2	Attenuates renal tubular epithelial injury and exerts renoprotective effects	As a mitophagy receptor, mediates mitophagy to clear damaged mitochondria, improves mitochondrial dysfunction, and inhibits NLRP3 inflammasome activation, thereby alleviating renal tubular epithelial cell injury.
PINK1/Parkin	Activates mitophagy, inhibits excessive inflammatory responses, and exerts renoprotective effects	As a key regulatory pathway of mitophagy, mediates the clearance of damaged mitochondria, improves mitochondrial function, and suppresses excessive inflammatory responses, thereby exerting renoprotective effects.
AGEs	Promote DKD progression	When degraded in renal tubules or excessively produced, they promote DKD progression.
BNIP3/NIX, FUNDC1	Attenuate renal tubular injury and improve renal function	Reduce ROS and inhibit ferroptosis; clear dysfunctional mitochondria under physiological conditions.
ROS	Injure renal tubular epithelial cells	Exacerbate ferroptosis; inhibit the mitophagy pathway.

**Table 2:** Roles of mitophagy in cell death.

Type of Cell Death	Role of Mitophagy	Effect on Cell Death	References
Apoptosis	Clears damaged mitochondria and reduces the release of pro-apoptotic factors	Inhibits apoptosis	DOI: 10.4062/biomolther.2023.149 [40]
Apoptosis	Impaired mitophagy leads to accumulation of damaged mitochondria	Promotes amplification of apoptotic signaling	DOI: 10.1080/13510002.2024.2404794 [41] DOI: 10.1038/s41419-025-07438-x [42]
Necroptosis	Excessive ROS production promotes complex formation	Promotes necroptosis	DOI: 10.7150/ijbs.83906 [43]
Pyroptosis	Clears damaged mitochondria and reduces the release of mtROS and oxidized mtDNA	Inhibits pyroptosis (by suppressing excessive NLRP3 activation)	DOI: 10.3389/fimmu.2026.1767802 [32]
Pyroptosis	Increased release of mtROS and oxidized mtDNA	Promotes pyroptosis	DOI: 10.3389/fimmu.2026.1767802 [32]

**Table 3:** Major roles of Ferroptosis in diabetic nephropathy.

Key Molecules/Targets	Roles	Mechanisms
ALOX15, HMOX1	Promote ferroptosis and aggravate DKD	Circulating levels of the ferroptosis execution factors ALOX15 and HMOX1 are significantly upregulated in DKD and are positively correlated with the key inflammatory mediators IL-1β, IL-6, and TNF-α.
IL-6	Promotes intracellular iron accumulation in renal tubular cells	Upregulates hepcidin via the JAK2/STAT3 pathway, promotes internalization and degradation of ferroportin, restricts iron efflux, and leads to intracellular free iron accumulation, thereby promoting ferroptosis.
TNF-α	Exacerbates intracellular iron overload	Upregulates divalent metal transporter 1 (DMT1), increases iron uptake, and causes intracellular iron overload, thereby promoting ferroptosis.
ROS	Promote ferroptosis and aggravate DKD	Attack polyunsaturated fatty acids, trigger lipid peroxidation, and produce MDA, thereby promoting ferroptosis.
NLRP3	Mediates renal tubular injury	In DKD characterized by proteinuria, the NLRP3 inflammasome promotes renal tubular injury and fibrosis.
CNPY2	Aggravates DKD-related renal tubular injury	CNPY2 overexpression accelerates ferroptosis and disrupts MAM integrity, leading to mitochondrial dysfunction and endoplasmic reticulum stress, thereby aggravating DKD-related renal tubular injury.

**Table 4:** Molecular Targets and Pathways of Antagonistic Effects of Mitophagy and Ferroptosis in Renal Tubular Injury.

Experimental Model	Target/Pathway	Effect on Mitophagy	Effect on Ferroptosis	References
AKI	ROS/HO-1/GPX4 axis	Promotes mitophagy	Inhibits ferroptosis in renal tubular cells	DOI: 10.7150/ijbs.80775 [82]
DKD	Sestrin2/AMPK/PGC-1α pathway	Promotes mitophagy	Inhibits ferroptosis	DOI: 10.1016/j.jep.2025.119579 [49]
DKD	PINK1-mediated mitophagy	Promotes PINK1-mediated mitophagy	Inhibits high glucose-induced ferroptosis in HK-2 cells	DOI: 10.1007/s10753-023-01940-0 [77]
DKD	Reactivation of high glucose-suppressed mitophagy	Reactivates mitophagy	Protects renal tubular cells from ferroptotic injury	DOI: 10.1080/10715762.2023.2277143 [78]
DKD	HIF-1α/MIOX signaling axis	Restores mitophagy	Inhibits ferroptosis	DOI: 10.1038/s41581-024-00889-z [4]
DKD	PINK1-mediated mitophagy; upregulated GPX4, SLC7A11, GSH	Promotes mitophagy in a PINK1-dependent manner	Alleviates high glucose-induced lipid peroxidation and ferroptosis	DOI: 10.1007/s00018-023-05078-y [5]

**Table 5:** Molecular Targets and Pathways of Synergistic Effects of Mitophagy and Ferroptosis in Renal Tubular Injury.

Model	Protein/Molecule/Pathway	Effect on Mitophagy	Effect on Ferroptosis	References
Pancreatic cancer	Myoferlin	Overactivation	Promotes lipid peroxidation and ferroptosis	DOI: 10.1016/j.redox.2022.102324 [11]
Patulin-induced AKI	3-MA	Pharmacological blockade of autophagy	Significantly attenuates ferroptosis	DOI: 10.1021/acs.jafc.1c08349 [84]
Cisplatin-induced AKI	IDH1-R132H mutation	Mitochondrial dysfunction	Enhances cisplatin-induced oxidative stress and ferroptosis	DOI: 10.1038/s41418-024-01381-8 [81]
DKD	PERK/ATF4/CHAC1 pathway	Mitochondrial dysfunction	↑Accompanied by enhanced ferroptosis	DOI: 10.1002/advs.202416441 [72]

## Data Availability

No new data were created or analyzed in this study. Data sharing is not applicable to this article.

## Abbreviations

The following abbreviations are used in this manuscript:

DKD	Diabetic kidney disease
PTECs	Proximal tubule epithelial cells
ATP	Adenosine Triphosphate
FAO	Fatty acid β-oxidation
AGEs	Advanced Glycation End Products
PINK1	PTEN-induced kinase 1
BNIP3	Bcl-2-interacting protein 3
FUNDC1	FUN14 domain-containing protein 1
GPX4	Glutathione Peroxidase 4
LIR	LC3-interacting regions
NCOA4	Nuclear Receptor Coactivator 4
FAM134B	Family with sequence similarity 134, member B
HIF-1α	Hypoxia-inducible factor 1α
SCF-FBXL4	SKP1-CUL1-F-box protein 4 (FBXL4) E3 ubiquitin ligase complex
CK2	Casein Kinase 2
MARCH5	Membrane-associated RING-CH-type finger protein 5
FIP200	FAK family kinase-interacting protein of 200 kDa
ULK1	UNC-51-like kinase 1
ROS	Reactive oxygen species
mtDNA	mitochondrial DNA
PHB2	Prohibitin 2
mtROS	mitochondrial reactive oxygen species
Bcl-2	B-cell lymphoma 2
MOMP	Mitochondrial outer membrane permeabilization
RIPK1	Receptor-interacting protein kinase 1
RIPK3	Receptor-interacting protein kinase 3
MLKL	Mixed lineage kinase domain-like protein
GSDMD	Gasdermin D
IL-1β	Interleukin-1β
IL-18	Interleukin-18
NLRP3	NOD-like receptor family pyrin domain-containing 3
AMPK	Adenosine Monophosphate-Activated Protein Kinase
SESN2	Sestrin2
SLC7A11	Solute Carrier Family 7 Member 11
GSH	Glutathione
ALOX15	Arachidonate 15-Lipoxygenase
HO-1	Heme Oxygenase 1
JAK2	Janus kinase 2
STAT3	Signal transducer and activator of transcription 3
DMT1	Divalent metal transporter 1
cGAS–STING1	Cyclic GMP-AMP Synthase–Stimulator of Interferon Genes 1
UCP2	Uncoupling protein 2
AK1–STAT1–SLC7A11	Janus kinase 1/signal transducer and activator of transcription 1/solute carrier family 7 member 11
MDA	Malondialdehyde
HK-2	Human renal proximal tubular epithelial cells
CNPY2	Canopy FGF signaling regulator 2
MAM	Mitochondrial-associated endoplasmic reticulum membrane
UHRF1	Ubiquitin-like with PHD and ring finger domains 1
MIOX	Myo-inositol oxygenase
NRF2	Nuclear factor erythroid 2-related factor 2
LIP	Labile iron pool
3-MA	3-methyladenine
IDH1-R132H	Isocitrate Dehydrogenase 1-Arginine to Histidine mutation at position 132
SQSTM1	Sequestosome-1
DN	Diabetic nephropathy
PGC-1α	Peroxisome proliferator-activated receptor gamma coactivator 1-alpha
RGN	Regucalcin
SMP30	Senescence marker protein-30
CD63	Cluster of differentiation 63
